# Beyond Age: Exploring Health Literacy, Well-Being, and Stress Management Among the Elderly

**DOI:** 10.1177/01939459261442785

**Published:** 2026-05-26

**Authors:** Joana Ferreira, Tânia Gaspar

**Affiliations:** 1 Lusofona University, SPIC, HEI-LAB, Lisbon, Portugal

**Keywords:** older adults, health literacy, digital health literacy, well-being, stress management, active aging

## Abstract

**Background:**

Aging can increase stress, making effective stress management strategies essential for promoting healthy aging. However, many older adults face difficulties because of low health literacy, which negatively impacts their well-being. Few studies have explored the relationship between health literacy, well-being, and stress management.

**Objectives:**

We aimed to examine relationships between health literacy (including digital health literacy), well-being, and stress management among older adults. Specifically, we sought to: (1) assess levels of health and digital health literacy; (2) examine differences according to age, gender, education, and senior university attendance; and (3) analyze the associations between health literacy, stress management, and well-being, including the potential role of stress management in this relationship.

**Methods:**

A quantitative cross-sectional study was conducted with 579 participants aged 65 to 98 years, of whom 64.1% were women.

**Results:**

Older adults with lower education levels and those who do not attend a senior university had lower levels of health literacy. Internet use for health-related purposes was also limited. Stress management skills fully mediated the relationship between both traditional and digital health literacy and well-being, indicating that higher health literacy is associated with better well-being through improved stress management.

**Conclusion:**

Strengthening health literacy can promote well-being by improving stress management. Interventions should include informational sessions, tailored educational materials, and skill-building workshops. Senior universities can play a key role by offering technology and health-related classes that empower older adults to access reliable online health information and improve their overall health knowledge.

## Introduction

Throughout life, human beings undergo various transformations, with old age marked by physical, emotional, and social changes that can negatively impact health and well-being. In this context, factors such as the promotion of active aging, stress management, and health literacy, including digital literacy, become essential for the autonomy, quality of life, and well-being of older adults.

### Health Literacy, Well-Being, and Stress Management in Old Age

Human beings undergo continuous change throughout life, marked by distinct stages and developmental milestones. The final stage, often the longest, is commonly referred to as “old age.”^1^ According to the World Health Organization (WHO), the chronological age at which an individual may be considered elderly depends on the country’s socioeconomic development, being 60 years for developing countries and 65 years for more developed countries. This corresponds to Erikson’s final psychosocial stage of development.^2^ This period is characterized by physical decline, increased vulnerability to illness (eg, dementia), and significant life transitions such as retirement and the loss of loved ones. During this stage, individuals may experience a decrease in motivation, self-esteem, and sense of belonging.^3^

Promoting opportunities that support physical and mental health, autonomy, and social participation contributes to active aging.^4^ Initiatives such as senior universities foster engagement through educational, cultural, social, and sports activities, helping reduce isolation and enhance the quality of life among older adults.^5^

The WHO defines health as complete physical, mental, and social well-being, not just the absence of disease.^6^ Well-being can be objective, measured by indicators like income, or subjective, encompassing emotional experiences and life satisfaction.^7,8^ Chronic disease, long-term and multifactorial conditions, such as cardiovascular and oncological diseases,^9,10^ and aging-related changes (eg, loss of abilities) can increase stress, interfering with daily tasks and overall well-being.^11,12^

According to the Transactional Model of Stress and Coping, stress can be defined as the relationship between an individual and their environment, where the individual assesses their resources to cope with their environment. If the situation is judged to be threatening and the resources are considered insufficient, it is perceived as stressful.^13,14^ It is through this continuous cognitive appraisal process that the same situation can be perceived as more stressful by one individual than by another, giving rise to the concept of perceived stress (ie, the subjective evaluation an individual makes of the level of stress they are experiencing at any given moment).^15^

Aging and its changes may be perceived as stressful, affecting well-being and health.^16^ Therefore, increasing the availability of information on how to manage stress is essential. Stress management techniques, including cognitive-behavioral programs, mindfulness,^17^ healthy lifestyle habits (eg, diet or physical), and community engagement,^18^ have been associated with increased well-being and quality of life and reduced stress. However, access to and understanding of information about stress management may depend on individuals’ health literacy levels.^19^

### Health Literacy

Health literacy is the ability to access, understand, evaluate, and apply health-related information to promote and maintain health and well-being,^20^ which enables individuals to effectively navigate healthcare, disease prevention, and health promotion.^21^ Low health literacy is associated with poorer health outcomes, increased hospitalization, and difficulty managing illness.^22^ Therefore, health literacy is a crucial skill for older adults. However, older adults are one of the groups more vulnerable to low levels of both traditional and digital health literacy.^23,24^

In the digital era, digital health literacy extends these competencies to online environments and the use of electronic devices to find and apply health-related information. Digital health literacy encompasses a combination of analytical (ie, traditional and numeracy, media and information literacy) and context-specific literacies (ie, health, scientific, and computer literacy), and it is shaped by personal, social, and environmental factors.^25^ Although improving digital health literacy may enhance autonomy and well-being, older adults often face barriers such as limited technological experience.^26^

Both distal (social, environmental) and proximal (personal, situational) factors influence health literacy.^21^ Age^19,27^ and lower education levels^28,29^ are consistently associated with lower traditional and digital health literacy. Gender findings are mixed, with some studies showing that men have lower literacy^30^ while others find no differences.^31,32^ Senior university attendance has been associated with higher levels of traditional and digital health literacy.^33^

Health literacy influences participation in healthcare, autonomy, health behaviors (eg, stress management), and health outcomes, including well-being.^21^ Literature suggests that higher levels of traditional and digital health literacy are associated with greater well-being.^34^ As for the relationship between health literacy and stress management, findings are mixed, as some studies indicate the existence of a significant association^35^ while others found no associations at all.^36^

### Theoretical Framework

Andersen’s Behavioral Model of Health Service Use provides a comprehensive theoretical framework to understand how individual and contextual factors influence health behaviors and outcomes. In this study, the model is used to guide the conceptual understanding of how health literacy, both traditional and digital, acts as an enabling resource that facilitates the adoption of health-promoting behaviors, such as stress management strategies, and ultimately influences perceived health and well-being. Within this framework, individual characteristics (eg, predisposition skills such as health literacy) influence health outcomes (eg, perceived health status) through health-related behaviors (eg, personal health practices), which are, in turn, correlated with both health outcomes and individual characteristics.^37,38^

The literature has established that the various changes occurring throughout aging can impact stress levels in older adults. Higher stress levels can significantly affect both the physical and psychological health of older individuals, making it crucial to find ways to prevent health deterioration and promote better well-being.^16^ However, despite these findings, few studies have comprehensively examined the role of stress management as a mediator between both traditional and digital health literacy and overall well-being in older adults. Thus, considering the information previously presented, the current study aims to explore how both traditional and digital health literacy interact with stress management capacities and, subsequently, well-being, in older adults.

## Purpose

Based on the information presented, the following research question was formulated: Is there a relationship between health literacy (including digital health literacy), well-being, and stress management in the aging process? The main objective was to characterize the relationship between health literacy (including digital), well-being, and stress management in older adults. The specific objectives were as follows: (a) to assess the level of health literacy (both traditional and digital) in older adults; (b) to compare levels of health literacy (traditional and digital) according to age, gender, education level and attendance to a senior university; (c) to analyze the relationship between health literacy (traditional and digital) and well-being in older adults; (d) to analyze the relationship between health literacy (traditional and digital) and stress management in older adults; and (e) to gain a deeper understanding of the potential relationship between health literacy (including digital health literacy), stress management, and well-being in older adults.

## Methods

### Design and Participants

This was a quantitative, cross-sectional study. Inclusion criteria comprised individuals aged 65 years or older. The healthcare staff helped identify individuals who were cognitively able to complete the questionnaire, excluding those with dementia or other conditions that could compromise understanding. Data collection took place between October 2023 and March 2024.

#### Sample Size Estimation

To estimate the sample size, the approximate number of people aged 65 years and older in Portugal was considered, which in 2023 was around 2 800 000. Using a scientific sample size calculator, with a 95% confidence level and a 5% margin of error, the required minimum sample size was estimated to be approximately 385 participants. Therefore, the total sample of 579 participants meets the recommended requirements.

### Instruments

#### Sociodemographic Variables

A sociodemographic questionnaire was developed to gather relevant information about the participants, important both for sample characterization and data analysis. The main data collected relate to age, gender, marital status, education level, area of residence, employment status, presence of chronic disease, and participation in senior university programs.

#### Health Literacy

To measure this variable, the Health Literacy Survey 19 (HLS_19_-Q12) was used.^39,40^ This is a short form of the original HLS_19_-Q47 and includes 12 items assessing overall health literacy, covering the dimensions of health promotion, disease prevention, and healthcare (eg, “How easy would you say it is to make decisions to improve your health and well-being?” [item 12]). Responses are rated on a five-point scale from “Very easy” (4) to “Don’t know” (0), with higher scores indicating greater health literacy. Qualitative classification of health literacy levels can be achieved by converting the obtained scores to a percentage scale ranging from 0 to 100, allowing categorization as “inadequate” (below 50), “problematic” (50-66.66), “adequate” (66.67-83.33), and “excellent” (above 83.34). Cronbach’s alpha values on the original scale^39^ ranged between 0.67 and 0.87. In the current sample, the internal consistency of the whole instrument was excellent with a Cronbach’s alpha of 0.92. Cronbach’s alpha values for the 3 subscales were as follows: health promotion, α = 0.82; disease prevention, α = 0.82; and healthcare, α = 0.79.

#### Digital Health Literacy

To measure this variable, the eHealth Literacy Scale (eHEALS) was used.^41,42^ It consists of 8 items (eg, “I know what health resources are available on the Internet” [item 1]) that assess experience in using the Internet to obtain health information. It has a 5-point Likert scale ranging from “Strongly disagree” (1) to “Strongly agree” (5). Higher scores indicate a higher level of digital health literacy. Consistent with other studies conducted in similar populations, the cutoff value was set at 26, with scores above 26 indicating higher levels of digital health literacy and scores below 26 indicating lower levels of digital health literacy.^43^ Cronbach’s alpha values on the original scale^41^ were around 0.88. In the present sample, Cronbach’s alpha for the total scale was 0.97, indicating good internal consistency.

#### Well-Being

The World Health Organization-5 Well-Being Index (WHO-5)^44,45^ was used to assess well-being over the past 2 weeks. It includes 5 items (eg, “I have felt cheerful and in good spirits” [item 1]) using a 6-point Likert scale ranging from “All the time” (5) to “At no time” (0). The total score, ranging from 0 to 25, is obtained by summing up the 5 items, with higher scores indicating better mental well-being. To express the result as a percentage between 0 and 100, the raw score is multiplied by 4. A percentage score below 50 (or a raw score below 13) has been proposed as a threshold indicating poor mental well-being. In the present sample, Cronbach’s alpha for the total scale was 0.89, indicating good internal consistency.

#### Stress Management

The Perceived Stress Scale (PSS)^15,46^ was used to assess the degree to which an individual evaluates their life situations as stressful and their stress management skills. While the PSS-4 was originally designed to measure perceived stress, previous research has also used it as an indicator of individuals’ perceived capacity to manage stress, with higher scores reflecting a more positive perception of stress management.^47^ The version used in this study consists of 4 items (eg, “In the last month, how often have you felt you were unable to control the important things in your life?” [item 1]), with a 5-point Likert scale ranging from “Never” (0) to “Very often” (4). To calculate the score, the values of each item are summed, resulting in a total score ranging from 0 to 16. Items 1 and 4 were reverse-coded, as in previous studies; higher scores indicate lower perceived stress, meaning a better ability to manage stress. Some studies reported that PSS-4 does not have cutoffs but is instead compared to normative data.^48^ In the original 4-item scale^15^ Cronbach’s alpha was 0.72. In the current sample, Cronbach’s alpha was 0.73, indicating good internal consistency.

### Procedures

#### Ethical Considerations

The present study was approved by the Comissão de Ética e Deontologia para a Investigação Científica (CEDIC) of the Escola de Psicologia e de Ciências da Vida, Universidade Lusófona de Humanidades e Tecnologias (CEDIC-2022-07-07).

#### Participant Recruitment

Participants were recruited using a convenience sampling approach, both online and in person. The study information was shared through emails and social media, targeting senior associations and universities. A general online link was available for all potential participants. In-person recruitment was concentrated in the Lisbon Metropolitan Area and the Autonomous Region of the Azores, where the research team could more easily encourage participation in day centers, senior associations, and residential care facilities, with additional participation from all other regions of Portugal. To include individuals without Internet access, participants were recruited through retired persons’ associations and day centers, where questionnaires were administered on paper or via interviews as needed.

#### Data Collection

Researchers and staff helped ensure comprehension without influencing responses. Standardized instructions were provided to all participants, and research assistants were trained to maintain consistency across administration formats. All participants received the same written and/or verbal standardized instructions before beginning the questionnaire. For in-person administration, questionnaires were completed in separate and quiet rooms, with assistance available to support comprehension when required. Online participants completed the same questionnaire independently. Written informed consent was obtained from all participants, ensuring voluntary participation, confidentiality, and understanding of the study procedures. Questionnaires concluded with a thank-you message.

### Data Analysis

Analyses were conducted using IBM SPSS Statistics (version 26.0; IBM Corp., Armonk, NY, USA). The database was first prepared, including missing value coding and reverse scoring of the PSS items to ensure higher values reflect better stress management. Descriptive statistics, reliability analysis (Cronbach’s α), and normality tests were conducted. Sample distribution was examined through skewness and kurtosis analysis along with the Kolmogorov-Smirnov test, based on established criteria.^49^ To address the second objective, 2 statistical procedures were applied: an independent samples *t*-test to compare groups based on gender and participation in senior universities, and an analysis of variance to evaluate differences across age groups and education levels, using Duncan post-hoc tests where appropriate. For the third and fourth objectives, Pearson’s bivariate correlation was used to explore associations between variables, following Cohen’s guidelines for interpreting effect sizes.^50^ To address the fifth objective, a mediation analysis (Model 4) was conducted using the PROCESS Macro for SPSS.^51^

## Results

### Participant Characteristics

Initially, 684 participants answered the questionnaire, but 105 were excluded for not meeting the age criteria. In total, 579 participants completed the questionnaire, aged between 65 and 98 years (M = 74.1, SD = 6.7), with women representing the majority of the sample. Detailed sociodemographic characteristics are presented in Table 1.

**Table 1.** Sociodemographic Characteristics of the Sample (N = 579).

Variable	n (%)
Chronic illness	
Yes	327 (56.5)
No	252 (43.5)
Marital status	
Single	38 (6.6)
Married/Cohabiting	293 (50.6)
Divorced/Separated	68 (11.7)
Widowed	180 (31.1)
Education level	
Did not complete basic education	101 (17.4)
Basic education completed	193 (33.3)
Secondary education completed	137 (23.7)
Bachelor’s degree	98 (16.9)
Master’s degree	21 (3.6)
Doctorate	13 (2.2)
Other	16 (2.8)
Senior university participation	
Yes	158 (27.3)
No	403 (69.6)
Yes, but no longer attend	18 (3.1)
Residence	
North	19 (3.3)
Porto Metropolitan Area	20 (3.5)
Center	62 (10.7)
Lisbon Metropolitan Area	328 (56.6)
Alentejo	6 (1.0)
Algarve	5 (0.9)
Autonomous Region of Madeira	5 (0.9)
Autonomous Region of Azores	134 (23.1)
Employment status	
Retired	521 (90.0)
Retired with professional activity	44 (7.6)
Other	14 (2.4)

Age, M = 74.1, SD = 6.7.

### Descriptive Statistics of Health Literacy

The sample in this study presents a satisfactory average health literacy score of 33.8 (SD = 8.2). To qualitatively classify the mean level of health literacy, the score was converted to a scale ranging from 0 to 100, resulting in a value of 70.89, which indicates an adequate level of health literacy.^40^ Participants reported greater ease in accessing professional help and understanding advice from healthcare providers, whereas more difficulties were observed in items related to mental health literacy. Detailed descriptive statistics are presented in Table 2.

**Table 2.** Descriptive Statistics of Health Literacy (N = 579).

Items	M	SD	% Say it is easy
.. to find out where to get professional help when you are ill?	3.7	0.82	94.1
. . . to act on advice from your doctor or pharmacist?	3	0.83	87.2
. . . to understand advice concerning your health from family or friends?	3	0.77	86.0
. . . to judge how your housing conditions may affect your health and well-being?	2.9	0.91	81.3
. . .to judge if information on unhealthy habits, such as smoking, low physical activity or drinking. too much alcohol, is it reliable?	3.9	1.00	79.8
. . . to find information on healthy lifestyles such as physical exercise, healthy food or nutrition?	2.8	0.95	76.3
. . . to make decisions to improve your health and well-being?”	2.8	0.90	73.9
. . . to understand information about what to do in a medical emergency?	2.7	0.92	70.5
. . .to decide how you can protect yourself from illness using information from the mass media?	2.6	0.98	66.1
. . . to understand information about recommended health screenings or examinations?	2.6	1.03	66.0
. . . to judge the advantages and disadvantages of different treatment options?	2.5	1.0	58.7
. . . to find information on how to handle mental health problems?	2.1	1.1	45.3

Abbreviations: M, mean; SD, standard deviation.

### Descriptive Statistics of Digital Health Literacy

Regarding digital health literacy, the sample showed low levels (M = 19.4, SD = 8.9), with more than half of the participants presenting a low level of digital health literacy. Less than half of the participants reported ease in using the internet for health-related purposes, particularly regarding evaluating the quality of online information and feeling confident in using such information for health-related decisions. Detailed descriptive statistics are presented in Table 3.

**Table 3.** Descriptive Statistics of Digital Health Literacy (N = 579).

Items	M	SD	% Agrees
I know how to use the Internet to answer my health questions	2.5	1.3	32.1
I know how to use the health information I find on the Internet to help me	2.5	1.2	29.2
I know where to find helpful health resources on the Internet	2.5	1.3	29
I know what health resources are available on the Internet	2.4	1.1	23.7
I know how to find helpful health resources on the Internet	2.4	1.2	28.2
I can tell high-quality from low-quality health resources on the Internet	2.4	1.3	25.2
have the skills I need to evaluate the health resources I find on the Internet	2.4	1.2	25.6
I feel confident in using information from the Internet to make health decision	2.2	1.3	21.6

Abbreviations: M, mean; SD, standard deviation.

### Group Comparisons

#### Gender

The results presented in Table 4 show that there are no statistically significant differences in traditional health literacy considering gender (*t*_[574]_ = −0.064, *P* = .949). However, there are statistically significant differences in digital health literacy (*t*_[574]_ = −2.920, *P* = .004, *d* = −0.245), with men showing higher digital health literacy scores (M = 20.8, SD = 8.9) than women (M = 18.5, SD = 8.9).

**Table 4.** Comparison of Traditional and Digital Health Literacy Values by Sex.

Health literacy type	Women (n = 369)		Men (n = 207)		*t*	*P*	*d*
	M	SD	M	SD			
Health literacy	33.8	8.2	33.9	8.4	−0.064	.949	
Digital health literacy	18.5	8.9	20.8	8.9	−2.920	.004	−0.254

#### Age Groups

To examine potential differences in health literacy and digital health literacy by age, the variable “Age” was divided into 3 groups following the age stratifications reported by PORDATA (Database of Contemporary Portugal): 65 to 69 years, 70 to 74 years, and ≥75 years.^52^ There are statistically significant differences among the 3 age groups in traditional health literacy (*F*_2_ = 15.429, *P* < .001, η^2^ = 0.051). According to Table 5, multiple comparisons revealed statistically significant differences between all groups, with the “65 to 69 years” group showing the highest average (M = 36.3, SD = 6.0), followed by the “70 to 74 years” group (M = 34.5, SD = 8.0) and, finally, the “75+ years” group (M = 32.1, SD = 8.4). Similarly, there were statistically significant differences in digital health literacy (*F*_2_ = 42.134, *P* < .001, η^2^ = 0.129), with multiple comparisons confirming statistically significant differences between all groups. The “65 to 69 years” group showed the highest average (M = 23.2, SD = 8.2), followed by the “70 to 74 years” group (M = 20.7, SD = 8.4), and finally, the “75+ years” group (M = 15.8, SD = 8.4).

**Table 5.** Comparison of Traditional and Digital Health Literacy Values, Considering Age Groups.

Health literacy type	65-69 years (n = 172)		70-74 years (n = 169)		75+ years (n = 238)		*F*	*P*	η^2^
	M	SD	M	SD	M	SD			
Health literacy	36.3	6.0	34.5	7.9	32.0	8.4	15.429	<.001	0.051
Digital health literacy	23.2	8.3	20.7	8.4	15.8	8.4	42.134	<.001	0.129

#### Educational Level

Statistically significant differences were found among all education groups in traditional health literacy (*F*_2_ = 61.428, *P* < .001, η^2^ = 0.180), as shown in Table 6. Multiple comparisons confirmed statistically significant differences among all groups. The “Higher Education” group had the highest average (M = 38.6, SD = 5.9), followed by the “Completed Compulsory Education” group (M = 36.3, SD = 5.6), and finally, the group that “Did Not Complete Compulsory Education” (M = 30.6, SD = 8.8). A similar pattern was found for digital health literacy, also showing statistically significant differences (*F*_2_ = 146.533, *P* < .001, η^2^ = 0.344), with the “Higher Education” group presenting the highest average (M = 26.1, SD = 7.3), followed by the “Completed Compulsory Education” group (M = 23.3, SD = 7.0), and finally, the group that “Did Not Complete Compulsory Education” (M = 14.4, SD = 7.3).

**Table 6.** Comparison of Traditional and Digital Health Literacy Values, Considering Education Level.

Health literacy type	Did not complete compulsory education (n = 294)		Completed compulsory education (n = 137)		Higher education (n = 132)		*F*	*P*	η^2^
	M	SD	M	SD	M	SD			
Health literacy	30.6	8.8	36.3	5.6	38.6	5.9	61.428	<.001	0.180
Digital health literacy	14.4	7.3	23.3	7	26.1	7	146.533	<.001	0.344

#### Senior University Attendance

There are significant differences in both traditional health literacy (*t*_[479.918]_ = −6.640, *P* < .001, *d* = −0.517) and digital health literacy (*t*_[362.645]_ = −4.010, *P* < .001, *d* = −0.350), based on whether participants attend a senior university. According to Table 7, participants attending a senior university had higher average scores in traditional health literacy (M = 36.7, SD = 5.9) and digital health literacy (M = 21.5, SD = 8.3) compared to those who did not attend, who scored lower in both traditional (M = 32.6, SD = 8.8) and digital health literacy (M = 18.4, SD = 9.0).

**Table 7.** Comparison of Traditional and Digital Health Literacy Values, Considering Attendance at a Senior University.

Health literacy type	Attending a senior university (n = 176)		Did not attend (n = 403)		*t*	*P*	*d*
	M	SD	M	SD			
Health literacy	36.7	6	21.5	8.3	−6.640	<.001	−0.517
Digital health literacy	32.6	8.8	18.4	9	−4.010	<.001	−0.350

### Correlations

The results presented in Table 8 show that there is a statistically significant, positive, and moderate correlation between traditional health literacy and digital health literacy (*r* = 0.451, *P* < .001). Traditional health literacy also positively correlates with well-being (*r* = 0.240, *P* < .001), though this is a weak correlation. Lastly, traditional Health Literacy and Stress Management are also significantly, positively, and moderately correlated (*r* = 0.335, *P* < .001).

**Table 8.** Correlations Between the Variables (N = 579).

Variable	Digital health literacy	Well-being	Stress management
Health literacy	0.451***	0.240***	0.335***
Digital health literacy		0.135**	0.285***
Well-being			0.541***

** *P* < .01; ***P < .001.

Digital health literacy is positively and significantly correlated with well-being (*r* = 0.135, *P* < .001) and with stress management (*r* = 0.285, *P* < .001), both being weak correlations. Finally, Stress Management and Well-Being are strongly and significantly positively correlated (*r* = 0.541, *P* < .001).

### Mediation Models

All regression models were tested and satisfied the standard linear regression assumptions required for analyses with the PROCESS macro. Residual independence was confirmed with Durbin-Watson statistics close to 2 in all models (ranging from 1.865 to 2.028), indicating no autocorrelation of errors. Diagnostic plots, including scatterplots of standardized residuals versus predicted values, revealed no evidence of nonlinearity or heteroscedasticity, as the residuals were randomly distributed. Variance inflation factor values ranged from 1.000 to 1.123 across models, confirming the absence of multicollinearity. The assumption of normally distributed residuals was met, as indicated by histograms approximating the normal distribution and P-P plots in which residuals closely followed the diagonal. Collectively, these results confirm the robustness and validity of the regression analyses underpinning the mediation models.

**Figure 1.**
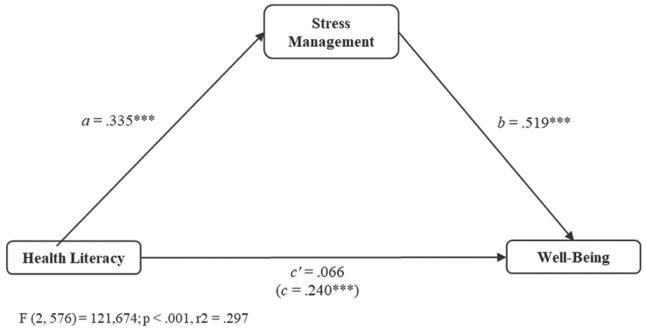
Mediation model regarding health literacy. *a* = effect of health literacy on stress management; *b* = effect of stress management on well-being; *c* = total effect of health literacy on well-being; *c′* = direct effect of health literacy on well-being, controlling for stress management. The indirect effect is represented by *a* *×* *b*.

The results showed a significant total effect between Health Literacy and Well-Being (*B* = 0.240, *P* < .001), as well as a statistically significant relationship between Health Literacy and Stress Management (*B* = 0.335, *P* < .001), and between Stress Management and Well-Being (*B* = 0.519, *P* < .001). When the variable Stress Management was included in the model, the direct effect was no longer significant (*B* = 0.066, *P* = .077). Thus, a full mediation was observed (*F*_2,576_ = 121.674, *P* < .001, *R*^2^ = 0.297), indicating that Stress Management Skills can be considered a mediator of the relationship between Health Literacy and Well-Being (bootstrapped 95% CI, 0.129-0.224). This mediation model can be seen in Figure 1.

**Figure 2.**
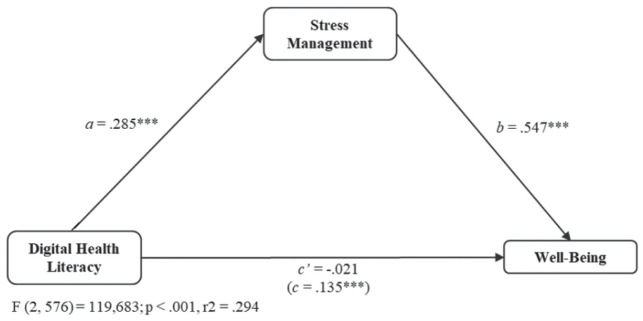
Mediation model regarding digital health literacy. *a* = effect of digital health literacy on stress management; *b* = effect of stress management on well-being; *c* = total effect of digital health literacy on well-being (simple regression coefficient); *c′* = direct effect of digital health literacy on well-being, controlling for stress management. The indirect effect is represented by *a* *×* *b*.

The results showed a significant total effect between Digital Health Literacy and Well-Being (*B* = 0.135, *P* < .001), as well as a statistically significant relationship between Digital Health Literacy and Stress Management (*B* = 0.285, *P* < .001), and between Stress Management and Well-Being (*B* = 0.547, *P* < .001). When the variable Stress Management was included in the model, the direct effect was no longer significant (*B* = −0.021, *P* = .564). Thus, a full mediation was observed (*F*_2,576_ = 119.683, *P* < .001, *R*^2^ = 0.294), indicating that Stress Management Skills can be considered a mediator of the relationship between Digital Health Literacy and Well-Being (bootstrapped 95% CI, 0.108-0.206). This mediation model can be seen in Figure 2.

## Discussion

The present study aimed to characterize the relationship between health literacy (including digital health literacy), well-being, and stress management among older adults. Overall, the findings indicate that higher levels of health literacy are associated with better stress management abilities, which in turn contribute to higher levels of well-being. These results reinforce health literacy as an important determinant of health and quality of life in older age.^21^

Participants demonstrated satisfactory levels of traditional health literacy, which may reflect a positive trend compared to similar studies.^53^ Family, friends, and healthcare professionals play a crucial role in helping older adults understand and navigate health information, contributing to the promotion of health literacy levels.^54,55^ However, difficulties in seeking mental health–related information were reported, suggesting lower levels of mental health literacy, which may hinder help-seeking behaviors, as observed in previous studies.^56^ In contrast, digital health literacy was low, consistent with national data,^33^ reflecting persistent age-related barriers in digital skills despite increased internet access.^57,58^

No significant gender differences were found in traditional health literacy, aligning with some previous studies.^31^ The absence of differences may be partially explained by the widespread dissemination of health information during the COVID-19 pandemic (eg, disease prevention and health promotion campaigns), which may have reduced gender disparities in access to health-related knowledge.^59^ However, men showed higher levels of digital health literacy, consistent with previous findings^60^, which may be linked to greater exposure to technology during their working lives (ie, computer engineering or internet-related jobs), allowing digital skills acquired at work to be maintained and effectively used in retirement.^60,61^

Age and education were significant factors, supporting previous literature showing that younger^24,27^ and more educated participants^28,29^ demonstrated higher levels of both traditional and digital health literacy. Educational opportunities across the life course may explain these results, as lower levels of education tend to be associated with lower literacy, which is essential for accessing, understanding, and applying health information.^62^ Younger individuals tend to feel more confident in accessing, understanding, and using health information, likely due to greater educational opportunities.^29^ Furthermore, older adults with more formal education seem to maintain higher digital literacy, further enhancing their health literacy.^63^

Participants attending senior universities showed higher levels of both traditional and digital health literacy, consistent with prior literature.^33^ These institutions offer learning opportunities, including health-related and computer courses, which help older adults develop important skills. Thus, senior universities may play an essential role in promoting health literacy in older adults.^5,64^ Although senior universities are common in Portugal, their mechanisms can be generalized to other lifelong learning settings, such as community centers. These institutions offer learning opportunities, social and recreational activities that help older adults develop health-related knowledge, digital skills, psychosocial resources that help prevent cognitive decline, and maintain physical autonomy and independence. By fostering health literacy, they support healthier behaviors, including regular exercise, which contribute to disease prevention, cognitive health, and overall well-being.^65^

Health literacy was positively associated with stress management and well-being. Individuals with higher health literacy may be better equipped to understand and apply health information in challenging situations,^36^ adopt healthier behaviors and manage their health more effectively, contributing to higher well-being.^66^

The mediation model showed that stress management mediates the relationship between health literacy and well-being. Higher health literacy facilitates access to and use of information, adherence to healthy behaviors, reducing engagement in risky behaviors,^67,68^ and enables the early identification of stress and the use of effective coping strategies, whereas limited health literacy may hinder these processes and contribute to higher stress levels.^69,70^ Adaptive stress management strategies, such as mindfulness, psychological support, physical exercise, and social engagement, can serve as protective factors in older age, contributing to improved health and well-being.^71,72^

Recent literature applying Andersen’s Behavioral Model increasingly emphasizes health literacy as an individual characteristic that shapes health behaviors and outcomes.^73,74^ This theoretical perspective aligns with the present study, which examines how traditional and digital health literacy influence well-being through stress management. Health literacy can be seen as an individual characteristic, as it symbolizes how older adults interact with health information, from seeking it to applying it. Higher health literacy enables older adults to understand and apply health information, make informed decisions, adopt health-promoting behaviors, and manage stress effectively, enhancing autonomy and overall well-being. Therefore, Andersen’s Behavioral Model of Health Service Utilization provides a framework for interpreting the observed relationships among health literacy, stress management, and well-being, supporting the view that enabling skills and individual characteristics may influence health outcomes through behavioral and cognitive pathways.^37,38^

### Limitations

Nevertheless, the present study has some limitations, such as the use of self-report instruments, which may increase the risk of social desirability bias, and the reliance on a convenience sample drawn from specific Portuguese regions limits the generalizability of the findings. Although all regions of Portugal participated in the study, the Lisbon Metropolitan Area and the Autonomous Region of the Azores had the highest number of participants due to the research team’s convenience. It is therefore important to clarify the contextual characteristics of these locations to facilitate understanding and enhance the potential for international generalization of the findings. These 2 areas differ in terms of population density, with the Lisbon Metropolitan Area being more densely populated than the Azores. Likewise, as an archipelago, the Azores are geographically more isolated and characterized by a more rural-urban mixed context compared to the Lisbon Metropolitan Area. Moreover, the Lisbon Metropolitan Area has a more extensive network of hospitals, health clinics, and senior universities than the Azores. Thus, although most participants were concentrated in these 2 regions, which may limit the generalizability of the results, the distinct characteristics of both areas contribute to the richness and diversity of the data. Future research should consider using probabilistic sampling methods to enhance representativeness.

Although the present study was conducted after the most critical phase of the COVID-19 pandemic, the lasting effects of the pandemic may help explain the relatively high levels of health literacy observed among older adults. It may be valuable to explore the long-term impact of the COVID-19 pandemic on health literacy levels among older adults, as well as the potential influence of individuals’ former occupations on their health literacy. Moreover, although the mediation model provided valuable insights into the indirect relationships among health literacy, health behaviors, and perceived health, its interpretation is limited by the cross-sectional nature of the data. Finally, this is not a longitudinal study; therefore, the conclusions and results should be interpreted with caution.

### Implications for Practice

Health professionals, such as psychologists and nurses, play a fundamental role in promoting health literacy among older adults, with an increasing emphasis on the importance of multidisciplinary collaboration. The findings of the present study may inform the work of psychologists, who can contribute to improving health literacy in this age group through informative sessions on the aging process, stress, and its management, and training focused on developing information-seeking skills.^75,20^ At the same time, it is essential to reinforce the role of nurses who, through both planned and spontaneous interactions with older adults, hold a privileged position in the transmission of health-related knowledge. Nurses act as facilitators and educators in addressing the limited health literacy needs of patients and contributing to the improvement of their overall health status. They play a key role in enhancing patients’ health literacy skills by supporting their ability to find, understand, evaluate, and apply health information related to their conditions.^76^ Based on these results, it may be beneficial to create counseling centers and additional training programs related to stress and stress management skills, as well as promoting a healthy lifestyle in older age (eg, healthy eating and physical activity). In this context, interdisciplinary collaboration among healthcare professionals is crucial for the effective promotion of health literacy, as it directly influences individuals’ capacity to understand, engage with, and manage their physical and mental health. Therefore, health literacy should be regarded as a core component in the promotion of healthcare and well-being, particularly among older adults, given their greater vulnerability associated with higher rates of chronic disease comorbidity.^20,77^

The results also demonstrate the importance of promoting digital health literacy in older adults, due to its contribution to well-being through stress management skills. The literature has shown that interventions designed to promote digital health literacy in older adults can be effective. They improve self-efficacy in computer use, making older adults more confident in their use. In this way, they also impact older adults’ knowledge in identifying health problems and their ability to seek information online and make informed decisions. This positively affects disease management capacity. Health professionals, such as psychologists and nurses, can adapt existing interventions from the literature to the needs of the older adults they work with. These interventions may be applied either in-person or online, each with its advantages (eg, in-person interventions allow professionals to pay more attention to older adults’ needs and adjust accordingly; online interventions remove time and space constraints). It becomes essential to raise awareness among older adults about the benefits of the internet for health matters. Health professionals should seek to stimulate older adults’ enthusiasm for learning to use electronic devices. Training skills in groups with similar levels of digital ability may also be beneficial, as well as paying attention to the continuous decline in the cognitive and physical functions of older adults.^57,78^

## Conclusions

Older adults with lower education and no participation in senior universities showed lower traditional and digital health literacy, along with limited internet use for health-related purposes. Health literacy was positively linked to well-being, particularly through better stress management. Overall, both forms of health literacy are essential for helping older adults navigate and manage their health, especially given their greater vulnerability to chronic conditions and age-related challenges.
